# The Associations of Emotional Intelligence, AI Self-Efficacy, and AI Literacy Among Nursing Undergraduates Under the NUR.S.E.S. Framework: Network Analysis

**DOI:** 10.2196/90253

**Published:** 2026-06-24

**Authors:** XiaoHui Fan, Jingjing Sun, LingHui Zhang, Jinrong Wang, BinHao Dong, XiaoHong Gao, Ruihua Jin, Panpan Huai

**Affiliations:** 1 Biomedical Research Center of Medical college, Xijing University, Xi'An, Shaanxi Province, China; 2 School of Nursing, Shanxi Medical University, 56 Xinjiannan Road, Yingze District, Taiyuan City, Shanxi Province, Taiyuan, Shanxi, 030001, China, 86 13130436162

**Keywords:** artificial intelligence literacy, emotional intelligence, AI self-efficacy, network analysis, nursing education

## Abstract

**Background:**

With the rapid development of generative artificial intelligence (AI) and its deep integration into nursing education, nursing students’ AI literacy (AS) has become a critical competency for their professional development. However, the patterns of associations among emotional intelligence (EI), AI self-efficacy (AILS), and AS in relation to comprehensive AS remain unclear.

**Objective:**

Based on the NUR.S.E.S. framework and using network analysis methods, this study systematically mapped the complex relational network among EI, AILS, and AS among undergraduate nursing students. It identified nodes with high centrality and bridging strength within this network, offering preliminary insights that may inform future educational interventions.

**Methods:**

A cross-sectional survey design was used, with 982 undergraduate nursing students from a university conveniently sampled in September 2025 as research participants. Assessments were conducted using the EI Scale, the AILS Scale, and the AS Scale. Using R (version 4.5.1; R Core Team), we constructed a Gaussian graph model, calculated centrality metrics such as node and bridge strength, and assessed network stability using the bootstrap method.

**Results:**

Network analysis showed that emotion regulation (strength centrality=1.355) and evaluative ability (strength centrality=1.323) showed the highest strength centrality, indicating their prominent positions within the network. Emotional perception (bridge strength=0.427) and comfort with AI (bridge strength=0.242) are the most critical bridge nodes, appearing to connect EI with AI technology systems. Simultaneously, the network architecture suggests that AILS may play a bridging role, effectively linking EI (particularly emotional perception as a bridging factor) with higher levels of AS.

**Conclusions:**

Cultivating AS among undergraduate nursing students is a system that deeply integrates emotional, cognitive, and technical confidence. EI was closely associated with AS, and AILS appeared to occupy a bridging position in the network. Educational interventions might consider enhancing emotional perception and comfort with AI, pending validation through longitudinal or experimental designs.

## Introduction

The rapid advancement of digital health care technologies, particularly the rise of generative artificial intelligence (GenAI) represented by large language models, is profoundly reshaping the practice paradigms and knowledge boundaries within health care [1]. For nursing students who will become the backbone of the future health care system, this represents both an unprecedented opportunity and a formidable challenge to their capabilities [2]. GenAI can be broadly defined as a subset of artificial intelligence (AI) that focuses on creating new, original, human-like content based on the data it is trained on [3]. A 2024 survey by the Digital Education Council reveals that as many as 85% of students frequently use GenAI in their academic work, with ChatGPT (OpenAI) being the most widely adopted platform among them [4]. Another global survey conducted in 2025 across 109 countries, which collected 20,000 responses, similarly indicated that students are extensively using GenAI tools, with ChatGPT usage exceeding 70% [5]. In the face of the widespread adoption of GenAI among students, education must go beyond introducing technological tools. More crucially, we must ensure that future nursing professionals can critically understand, prudently apply, and rationally evaluate AI, thereby transforming technology into a genuine professional enabler [6]. This lays the foundation for nursing students to apply AI tools and technologies appropriately and proficiently.

The American Nurses Association (ANA) asserts that, against the backdrop of emerging AI technologies, nurses applying AI in health care practice require not only ethical guidance but also uphold the core values of care and compassion inherent to nursing [7]. AI literacy (AS) serves as the core competency and vital bridge for integrating ethical guidance and humanistic care into AI application practices. It is regarded as a key competency in nursing education [8]. These capabilities extend beyond merely mastering and applying AI technologies (such as clinical decision support systems and predictive analytics) to encompass the higher-order thinking skills required to independently critically evaluate and assess the outcomes they generate [9]. As the backbone of the future health care system, nursing students are key participants in shaping AI applications in nursing. Their level of AS directly determines whether they can lead the intelligent transformation in this field, thereby profoundly influencing the breadth and depth of their personal career development [10]. Therefore, cultivating AS among nursing students is of paramount importance [11].

However, the cultivation of AS is not an isolated process; it is deeply rooted in the complex psychological, cognitive, and emotional capacity system of individuals [12]. Research indicates that an increasing number of people are turning to GenAI for guidance on interpersonal relationships, self-awareness, and emotional regulation [13]. Emotional intelligence (EI) refers to the ability to recognize, understand, and manage one’s own emotions, while also understanding and empathizing with the emotions of others [14]. Higher EI levels are significantly associated with nursing students’ stress management, clinical decision-making, and subjective well-being [15]. Research indicates that the use of GenAI can effectively alleviate stress and improve mental health outcomes, while simultaneously enhancing engagement and satisfaction in learning environments [16]. Specifically in the field of nursing education, Ching and Ho [17] found that including AI virtual humans in gamified teaching methods can enhance nursing students’ EI. Derakhshan [18] indicates that AI-assisted teaching can enhance students’ emotional engagement and goal orientation. Deng and Chen [19] also found that AI-driven environments, by providing personalized feedback and adaptive pathways, enhance their emotional resilience by reducing frustration and boosting students’ confidence. These findings collectively reveal that EI not only provides the psychological foundation for students to engage in effective and healthy interactions with AI, but it may also profoundly influence the development and enhancement of their AS by affecting their confidence and initiative when confronting new technologies.

EI provides the intrinsic emotional foundation for nursing students to effectively use AI, while confidence in their mastery of AI technology relies on AI self-efficacy (AILS). AILS is defined as an individual’s belief in their ability to perform specific tasks, which directly influences nurses’ willingness to adopt AI and their actual use of it [20]. Unlike general self-efficacy, AILS in nursing AI applications manifests as nursing students’ confidence in mastering AI tools, overcoming technical challenges, and using them to solve clinical or learning problems [21]. This confidence in specific technical domains serves as the intrinsic motivation driving health care professionals to actively engage with, deeply explore, and persistently use AI tools [22]. Research indicates that enhancing nursing students’ self-efficacy is crucial for alleviating psychological distress, ultimately improving their professional well-being and the quality of their future care [23]. In AI-enabled learning environments, Wang et al [24] indicate that self-efficacy and university support significantly enhance students’ acceptance of AI-driven blended learning. Gao et al [25] further confirm that integrating AI chatbots into writing instruction can enhance students’ AI self-efficacy. Lyu and Salam [26] also indicate that AI-driven personalized learning can enhance students’ self-efficacy, motivation, and digital literacy. Additionally, high levels of AILS significantly predict nursing students’ willingness to adopt AI technologies and the depth of their usage, thereby enhancing professional growth and adaptability in the rapidly evolving health care field [8].

Thus, EI and AILS jointly constitute the 2 core psychological factors influencing the development of AS among nursing students. EI ensures nursing students maintain essential emotional insight and empathy when interacting with AI and applying it in patient care, thereby providing the necessary human touch to technological implementation. Meanwhile, AILS provides the psychological drive to explore and use AI technologies, motivating students to transform potential technological advantages into realistic nursing capabilities [17]. To systematically guide and cultivate this complex psychological capability system, this study introduces an emerging theoretical framework—the NUR.S.E.S. framework [17]. This framework, recently proposed by Hoelscher and Pugh [27], aims to establish a structured pathway for developing AS within the nursing profession. Compared to generic technology adoption models or general educational technology frameworks, its novelty lies in deeply integrating the development of AS with the humanistic care, ethical responsibility, and clinical leadership inherent to the nursing profession, thereby better aligning with the contextual needs of nursing education. It is an acronym representing 6 core components:

- Navigate AI basics: promote understanding of AI fundamentals as a leadership responsibility critical to patient safety.
- Use AI strategically: purposefully apply AI tools to enhance care quality, safety, and outcomes.
- Recognize AI pitfalls and guide balanced approaches to innovation and risks such as bias, overreliance, and inaccuracy.
- Skills support: advocating for skill enhancement and professional development to prepare for AI integration in nursing.
- Ethics in action: guiding ethical AI use in nursing by promoting transparency, fairness, and accountability.
- Shape the future: directing AI implementation toward outcomes that align with nursing values and advance care equity.

This framework provides nursing educators with a forward-looking theoretical foundation rooted in nursing values, guiding students to develop their humanistic care, critical thinking, and ethical judgment capabilities simultaneously while mastering technical skills. This study used the NUR.S.E.S. framework, marking its first application in nursing education both domestically and internationally. Its internal structure clearly corresponds to core constructs such as EI (corresponding to the E and S dimensions), AILS (corresponding to the S dimension), and AS (corresponding to the N, U, R, and E dimensions), providing a structurally coherent and contextually relevant theoretical foundation for systematically analyzing the complex system formed by these variables.

As a cutting-edge, data-driven approach, network analysis transcends traditional linear assumptions to reveal intricate interactions among variables and precisely pinpoint their relative importance within a system [21]. This method provides a unique, systematic perspective for visualizing and quantifying the dynamic relational structure among EI, AILS, and AS [28]. By analyzing the interrelationships among variables, network analysis not only maps each measurement indicator to the theoretical dimensions of the NUR.S.E.S. framework but also tests whether the framework’s predefined dimensional divisions align with the actual patterns of variable associations observed in the data. Specifically, it examines whether variables within the same framework dimension are closely interconnected in the relational network and whether there are significant, unexpected associations between different dimensions, thereby providing empirical support for the framework’s validity [29].

Therefore, the objective of this study is to systematically analyze the complex network relationships and underlying mechanisms among EI, AILS, and AS based on the NUR.S.E.S. framework. By constructing a psychological network model, this study aims to identify factors that are associated with nursing students’ AS, thereby providing precise targets for educational interventions. We plan to conduct a survey of nursing students at a Chinese university to gain an in-depth understanding of their practical application of AI, including EI, AILS, and AS. By analyzing these findings, we aim to identify existing challenges and provide crucial theoretical foundations and practical guidance for cultivating future nursing professionals who can master intelligent technologies while upholding humanistic care.

## Methods

### Objects and Methods

A total of 982 full-time undergraduate nursing students from a particular university were selected as research participants. Among them, 117 were male, and 865 were female, with ages ranging from 18 to 24 years (mean age 21.18, SD 0.51 years). The inclusion criteria were (1) full-time undergraduate nursing students enrolled at the university; (2) students who had encountered or used AI-related technologies or tools in their daily studies or lives; and (3) voluntary participation in the experiment with signed informed consent and confidentiality agreements. Exclusion criteria applied to students on formal leave or extended sick leave during data collection. Participants could withdraw freely without affecting their studies or academic performance.

### Ethical Considerations

The research procedures have been approved by the Ethics Review Committee of Xijing University (Approval number: XJU-202502). Participants’ privacy has been protected. All participants provided written informed consent. No harm was inflicted upon participants. All nursing students had the right to choose whether to participate in this study. Data were securely stored and encrypted.

### Instruments

#### General Demographic Questionnaire

The research team independently developed a questionnaire to collect basic information. This survey aims to systematically examine key social-demographic factors that may influence research outcomes, primarily covering age, gender, home address, prior experience as a student leader, academic year, and average frequency of use of electronic devices and AI tools.

#### EI Scale

The EI Scale developed by Schutte et al [30] was used to assess students’ EI levels. The Chinese version was revised by the Chinese psychologist Caikang [31]. This version has demonstrated good reliability and validity among Chinese students. This scale comprises 4 dimensions, including emotional perception (12 items), self-emotional management (8 items), managing others’ emotions (6 items), and emotional application (7 items), totaling 33 items. A 5-point Likert scale was used to score, ranging from “Strongly Disagree” to “Strongly Agree,” with values from 1 to 5. Higher scores indicate a higher level of EI among students. The Cronbach α coefficient for the full scale was 0.90, and the test-retest reliability Cronbach α coefficient was 0.78. In this study, the Cronbach α coefficient for the scale was 0.959.

#### AILS Scale

The AILS scale, developed by Wang et al [32], is used to assess users’ self-efficacy when using AI technologies and products. The scale comprises 4 dimensions, including assistance (7 items), anthropomorphic interaction (5 items), comfort with AI (6 items), and technical skills (4 items), totaling 22 items. Scoring uses a 7-point Likert scale from “Strongly Disagree” to “Strongly Agree,” with values ranging from 1 to 7. Higher scores indicate greater levels of AILS among users. Confirmatory factor analysis resulted in *χ*²_196_=1.984; comparative fit index (CFI)=0.941; Tucker-Lewis index (TLI)=0.930; root-mean-square error of approximation (RMSEA)=0.079; and standardized root-mean-square residual (SRMR)=0.071. Cronbach α coefficient in this study was 0.953.

#### AS Scale

The AS Scale was developed by Wang and Chuang [33]. The scale comprises 4 dimensions, including awareness (3 items), usage (3 items), evaluation (3 items), and ethics (3 items). It is based on a 7-point Likert scale ranging from “Strongly Disagree” to “Strongly Agree,” with values ranging from 1 to 7, respectively. Higher scores indicate greater AS among individuals. Confirmatory factor analysis yielded the following indices: CFI=0.99, TLI=0.99, GFI=0.98, RMSEA=0.01, SRMR=0.03. The Cronbach α coefficient for the full scale was 0.830, while the Cronbach α coefficient in this study was 0.864.

### Correspondence Table Between the NUR.S.E.S. Framework and Questionnaire Measurement Dimensions

Guided by the theoretical framework of NUR.S.E.S., this study establishes conceptual mappings between the 6 core competency dimensions of the framework (navigate, use, recognize, skills support, ethics in action, and shape the future) and their corresponding measurement variables. This mapping aims to structure the AS of nursing students as defined by the framework into measurable psychological and competency indicators, thereby providing a theoretical basis for subsequent network analysis and testing the intrinsic interrelationships among the framework’s components.

### Theoretical Rationale for the Mapping Between NUR.S.E.S. Components and Measured Variables

#### Overview

The mapping shown in Table 1 follows from the conceptual definitions of each NUR.S.E.S. component as originally articulated by Hoelscher and Pugh [27], combined with established theoretical and empirical literature in nursing education, EI, and self-efficacy research.

**Table 1.** Correspondence table between the NUR.S.E.S. framework and questionnaire measurement dimensions.

Framework letters	Representative of core variables	Dimensions corresponding to the questionnaire	Dimensional implications	Mapping dimensions
N	Navigate AI^a^ basics	Awareness	Basic understanding of fundamental AI concepts, application scenarios, and its impact on the nursing field.	“Navigate AI basics” emphasizes initial exploration and foundational understanding of the field of AI. Only by acquiring this foundational understanding can students freely explore the AI knowledge system; therefore, the “awareness” dimension has been selected to measure this ability.
U	Use AI strategically	Use	The ability to operate and apply specific AI tools in practical nursing education, research, or simulated scenarios.	“Use AI strategically” focuses on practical application. This dimension directly assesses students’ ability to apply AI as a tool in specific scenarios, reflecting the practical aspect of “use.”
R	Recognize AI pitfalls	Evaluation	Critically examine and evaluate the accuracy and reliability of AI-generated information.	“Recognizing AI pitfalls” requires critical thinking to identify the limitations and risks of AI. The “evaluation” dimension is precisely the core competency involved in assessing and discerning information, and it aligns closely with the requirement to identify flaws.
S	Skills support	Technical Skills Assistance	Technical skills: confidence in one’s ability to learn and master AI technology itself. Assistance: believe that AI can serve as an effective tool to assist in completing learning or work tasks.	“Skills support” comprises 2 aspects: first, confidence in mastering AI skills themselves (technical skills); and second, trust in AI as a supportive tool (assistance). These 2 dimensions together underpin students’ self-efficacy at the skills level.
E	Ethics in action Emotional Intelligence (EI)	Ethics, Emotional Perception, Self-emotion Management, Managing Others’ Emotions, Emotional Application	Ethics: understand and adhere to ethical standards such as privacy and fairness involved in the use of AI in nursing practice. Emotional perception: accurately recognize your own and others’ emotional states. Self-emotion management: the ability to regulate and control one’s own emotions. Managing others’ emotions: the ability to influence and regulate others’ emotions. Emotional application: use emotional information to aid in thinking and problem-solving.	“Ethics in action” emphasizes the application of ethics in real-world nursing settings. EI as a whole construct is a foundational capacity for ethical practice, including perceiving, managing, and utilizing emotions. The 4 subdimensions of EI (EI1-EI4) are collectively used to measure EI, not to be split into other letters of the NURSES framework. The network analysis treats these 4 subdimensions as nodes belonging to the same EI community, which supports their theoretical coherence. Therefore, mapping all 4 EI subdimensions to the E dimension reflects the role of EI as a holistic supporter of ethics in action, not a forced segmentation.
S	Shape the future	Comfort with AI, Anthropomorphic Interaction	Comfort with AI: feeling relaxed and at ease when interacting with AI, without anxiety or a sense of threat. Anthropomorphic interaction: tends to engage in natural communication with AI in a manner similar to human interaction.	“Shape the future” embodies students’ open and proactive approach to leading the development of AI in the field of nursing. Comfort with AI reflects their level of acceptance, while anthropomorphic interactions demonstrate their willingness to collaborate with machines; together, these 2 factors form the psychological foundation for actively shaping the future of nursing.

a AI: artificial intelligence.

#### Navigate AI Basics (N)

Hoelscher and Pugh [27] define this component as promoting understanding of AI fundamentals as a leadership responsibility critical to patient safety. In operational terms, foundational knowledge of AI concepts and applications constitutes the prerequisite for any further engagement with AI technologies [34]. Accordingly, the awareness dimension of the AS Scale [33], which assesses students‘ understanding of basic AI concepts, application scenarios, and potential impacts, serves as a direct measure of this component.

#### Use AI Strategically (U)

This component concerns the purposeful application of AI tools to enhance care quality, safety, and outcomes [27]. Strategic use implies not only familiarity with AI but the ability to operate and apply specific AI tools in practical nursing education, research, or clinical scenarios. The Use dimension of the AS Scale [33], which captures students’ self-reported ability to work with AI tools in task-specific contexts, directly corresponds to this component.

#### Recognize AI Pitfalls (R)

Hoelscher and Pugh [27] emphasize that balanced approaches to innovation must include recognition of risks such as bias, overreliance, and inaccuracy. This critical evaluative competency is captured by the evaluation dimension of the AS Scale [33], which assesses students' ability to critically examine and judge the accuracy and reliability of AI-generated information.

#### Skills Support (First S)

The skills support component advocates for skill enhancement and professional development to prepare for AI integration in nursing [27]. This component encompasses both the confidence in one’s ability to learn and master AI technology itself and the belief that AI can serve as an effective tool to assist in completing learning or work tasks. The AILS Scale [32] operationalizes these 2 facets through its technical skills and assistance dimensions, respectively, drawing on Bandura’s broader theory of self-efficacy as domain-specific confidence in one’s ability to execute courses of action required to achieve desired outcomes [20].

#### Ethics in Action (E)

This component guides ethical AI use in nursing by promoting transparency, fairness, and accountability [27]. Importantly, ethical action in nursing requires not only knowledge of ethical principles but also the emotional capacities that underpin moral sensitivity and compassionate care. A substantial body of nursing literature demonstrates that EI—the ability to perceive, understand, manage, and use emotions—is positively associated with ethical sensitivity and moral reasoning in both nursing students and practicing nurses [8,15,31,35]. For example, Liu et al [36] found that EI mediates the relationship between empathy and moral sensitivity in Chinese student nurses, suggesting that emotional competence is not separate from but foundational to ethical practice [1]. The Schutte EI Scale [30], which measures 4 dimensions of EI (perception of emotion, self-emotion management, managing others' emotions, and emotional application), was therefore selected to capture the emotional competence component of ethical action. These 4 subdimensions are treated as a single conceptual community in the network analysis, consistent with the holistic contribution of emotional competence to ethical practice.

#### Shape the Future (Second S)

The final component directs AI implementation toward outcomes that align with nursing values and advance care equity [27]. Proactive, leadership-oriented engagement with AI presupposes that nurses feel psychologically comfortable with AI technologies and are willing to engage with them in a natural, quasisocial manner. Positive affective attitudes toward AI, including low anxiety and high comfort, have been identified as psychological prerequisites for adoption and sustained use in health care and educational contexts [21,37,38]. The AILS Scale captures these prerequisites through its comfort with AI and anthropomorphic interaction dimensions [32], which assess relaxed, nonanxious engagement with AI and the tendency to interact with AI in a human-like, conversational manner. These dimensions do not directly measure leadership behaviors but rather the psychological foundation without which proactive shaping of AI’s future in nursing is unlikely to occur.

This mapping provides a theoretically grounded operationalization of the NURSES framework into measurable psychological and competency variables, enabling empirical examination of the interrelationships among its 6 components through network analysis. The mapping is not intended as an exhaustive or exclusive operationalization but as a starting point grounded in existing validated instruments and established theoretical links.

### Data Collection

Researchers (XF and JW) contacted class administrators at target institutions via social media (WeChat; Tencent Holdings Limited). After explaining the study objectives and procedures and obtaining permission, they joined the corresponding class groups. Study information was disseminated through these class groups. Potential participants voluntarily confirmed their participation online after reading the informed consent form. The research team screened applicants based on predetermined inclusion and exclusion criteria. Students meeting the criteria were invited to participate in the survey. To facilitate organization and enhance response efficiency, researchers distributed electronic questionnaire links uniformly in classrooms during after-school hours. Data collection occurred from September 25 to September 28, 2025, using the online survey platform “WJX” for questionnaire distribution and retrieval. Participants independently completed the questionnaire after clicking the link and could exit at any time during the process. Completing the full questionnaire took an average of 10‐15 minutes. A total of 986 questionnaires were distributed, with 982 returned. Platform settings ensured only fully completed questionnaires were recorded as valid submissions, eliminating incomplete submissions from being counted. Verification confirmed that returned questionnaires were valid. Data from 982 participants were ultimately analyzed, yielding a valid response rate of 99.59%.

### Data Analysis

Descriptive statistics were calculated using SPSS (version 25.0; IBM Corp), with means and SDs for continuous variables and frequencies (percentages) for categorical variables. All continuous variables underwent normality testing in this study, and the results indicated that their distributions were generally normal [39].

Network analysis was performed using R (version 4.3.0; R Core Team). In 2-tailed tests, *P<*.05 was considered statistically significant. A Gaussian graph model was selected to construct the network structure using the R package “*qgraph*” [37]. To reduce spurious correlations and obtain a simplified network, the graph minimum absolute contraction and selection operator was used. The graph minimum absolute contraction and selection operator implements regularization techniques to constrain small partial correlations to zero, effectively eliminating potential false positive edges and yielding sparse, interpretable network structures [40]. To avoid overfitting and determine optimal tuning parameters, an extended Bayesian information criterion model is used to select the best-fitting model. This approach ensures that only the most robust and meaningful connections are retained in the final network.

In a network, nodes represent variables, and edges between 2 nodes represent the unique relationship between them, controlling for the influence of other nodes. Red and blue edges denote negative and positive correlations, respectively. The thickness of the edges corresponds to the strength of the association; thicker edges indicate stronger correlations [41]. The R package “*qgraph*” is used to calculate the strength, proximity, and betweenness centrality of nodes in a network [37]. Strength is defined as the sum of the absolute values of the edge weights connecting a node to all other nodes. Previous studies have shown that strength is the most stable and interpretable centrality measure in networks [42]. Compared to other centrality measures such as closeness and betweenness centrality, strength is more suitable for evaluating the importance of nodes [43]. Therefore, this study adopts strength as the centrality metric, with nodes exhibiting the highest strength values being regarded as the most influential nodes within the network.

Additionally, the R package “*networktools*” was used to calculate bridging strength and identify potential bridging nodes [44]. Bridging strength refers to the sum of the absolute values of the edge weights connecting a node to other community nodes, indicating the node’s importance to other communities. Nodes with the highest bridging strength values are considered bridging nodes within the network. Bridging nodes connect multiple domains, meaning interventions targeting these nodes can simultaneously impact multiple domains, which maximizes the effectiveness of the intervention.

Use the R package “*mgm*” to calculate the predictability for each node [45]. Predictability is a metric that reflects the extent to which the variance of a specific node can be explained by all its neighbors in the network. Nodes with high predictability are susceptible to the influence of their neighboring nodes.

The R package “*bootnet*” assesses the accuracy and stability of networks [46]. First, the accuracy of edge weights (the number of bootstrap samples is 1000) was examined by calculating their 95% CIs using nonparametric bootstrapping. Second, the stability of the network model is assessed using the case deletion procedure and the correlation stability (CS) coefficient, which indicates the proportion of samples that can be excluded from the analysis while maintaining at least 0.7 correlation with the original sample. According to network analysis guidelines, the CS coefficient should not fall below 0.25, and a value above 0.50 indicates sufficient stability [47]. Finally, a self-assessment method was used to conduct a difference test, examining variations in edge weights, node strengths, and node bridge strengths.

## Results

### General Information for Undergraduate Nursing Students

A total of 982 graduate nursing students were included in the statistical analysis, with their characteristics listed in Table 2. The mean age of participants was 21.7 years, and the vast majority (865/982, 88.1%) of participants were female. Among participants, 511 out of 982 (52.0%) resided in rural areas. The number of students in the first, second, third, and fourth years was 246 out of 982 (25.1%) participants, 242 out of 982 (24.6%) participants, 251 out of 982 (25.6%) participants, and 243 out of 982 (24.7%) participants, respectively. Among the 982 undergraduate nursing students, daily active use of GenAI tools was reported as follows: 22 out of 982 (2.2%) participants rarely used them, 608 out of 982 (61.9%) participants used them several times weekly, 297 out of 982 (30.2%) participants used them 3‐5 times daily, and 55 out of 982 (5.6%) participants used them more than 5 times daily.

**Table 2.** General information on nursing undergraduates.

Statistical variable	Frequency, n (%)
Sex	
Male	117 (11.9)
Female	865 (88.8)
Age (years)	
<18	42 (4.3)
18-20	733 (74.6)
20-22	204 (20.8)
≥22	3 (0.3%)
Family residence	
Townships	471 (48.0)
Rural	511 (52.0)
Have you ever served as a student leader?	
Yes	479 (48.8)
No	503 (51.2)
Grade level	
Freshman year	246 (25.1)
Sophomore year	242 (24.6)
Junior year	251 (25.6)
Senior year	243 (24.7)
The average daily time spent using electronic devices (such as smartphones, computers, tablets, etc) is approximately (hours)	
<2	42 (4.3)
2-4	291 (29.6)
4-6	439 (44.7)
6-8	161 (16.4)
>8	49 (5.0)
On average, how often do you actively use AI^a^ tools (such as ChatGPT [OpenAI], Wenxin Yiyan [Baidu], Deepseek [DeepSeek Artificial Intelligence Co, Ltd], etc) each day?	
Hardly ever used	22 (2.2)
Several times a week	608 (61.9)
3-5 times daily	297 (30.2)
>5 times a day	55 (5.6)
What is your primary purpose for using AI tools?	
Learning (such as researching information, assisting with homework, etc)	929 (94.6)
Entertainment and Leisure (such as smart chatbots, image/music generation, and video/audio recommendations)	468 (47.7)
Daily life (such as planning itineraries and obtaining health advice)	615 (62.6)
Work efficiency (eg, report writing, PowerPoint [Microsoft Corp] creation, data analysis)	595 (60.6)
Social needs (such as helping edit Moments captions and recommending chat topics)	336 (34.2)
Others	12 (1.2)
How familiar do you consider yourself to be with AI technology?	
I have absolutely no idea.	16 (1.6)
I’ve heard of it but don’t know much about it.	117 (11.9)
General understanding	569 (57.9)
Fairly familiar	262 (26.7)
Very familiar	18 (1.8)

a AI: artificial intelligence.

### Descriptive Statistics of Variables (List in Table 3)

Descriptive statistics for nursing undergraduates’ EI, AILS, and AS are presented in Table 3. Normality tests confirmed that all variables were approximately normally distributed. Data were described using mean (SD). SDs fell within normal ranges, with no outliers. Notably, “Emotional Perception (EI1)” demonstrated the highest predictive power and served as the critical component of the entire network.

**Table 3.** Descriptive statistics of emotional intelligence, AI self-efficacy, and AI literacy variables among nursing undergraduates (n=982).

Variable		Name	Mean (SD)	Expected influence	Bridge expected influence	Predictability
Emotional intelligence		EI^a^	3.57 (0.55)			
Emotional perception		EI1	3.41 (0.54)	1.248	0.427	0.832
Self-emotion management		EI2	3.51 (0.56)	1.016	0.118	0.788
Managing others’ emotions		EI3	3.67 (0.61)	1.099	−0.006	0.801
Emotional application		EI4	3.68 (0.62)	1.355	−0.024	0.840
AI^b^ self-efficacy		AILS^c^	4.68 (0.78)			
Assistance		AILS1	4.55 (0.84)	1.075	0.129	0.627
Anthropomorphic interaction		AILS2	4.62 (0.83)	1.091	0.069	0.679
Comfort with AI		AILS3	4.96 (0.98)	0.991	0.242	0.686
Technical skills		AILS4	4.59 (0.92)	0.854	0.077	0.496
AI literacy		AS^d^	4.60 (0.59)			
Awareness		AS1	5.19 (0.95)	0.862	0.015	0.597
Usage		AS2	4.43 (1.12)	0.838	−0.030	0.545
Evaluation		AS3	4.83 (0.94)	1.323	−0.011	0.726
Ethics		AS4	3.94 (1.19)	0.837	−0.033	0.287

a EI: emotional intelligence.

b AI: artificial intelligence.

c AILS: AI self-efficacy.

d AS: AI literacy.

### Network Analysis of EI, AILS, and AS Among Nursing Undergraduates

**Figure 1.**
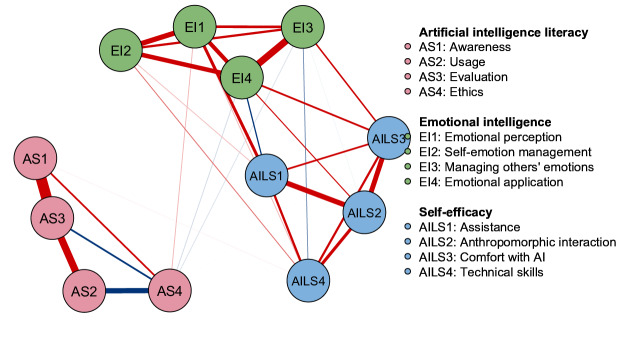
Network analysis of emotional intelligence (EI), AI self-efficacy (AILS), and AI literacy (AS) among nursing undergraduates. Node colors represent theoretical constructs: blue=AILS, green=EI, and red=AS. Letters on nodes denote dimension codes. Red indicates positive correlations; blue indicates negative correlations. Thicker lines denote stronger connections. AILS: artificial intelligence self-efficacy; AS: AI literacy; EI: emotional intelligence.

Figure 1 illustrates the network structure of EI, AILS, and AS among undergraduate nursing students. Node colors represent theoretical constructs: blue=AILS, green=EI, and red=AS. Letters on nodes denote dimension codes. Red indicates positive correlations; blue indicates negative correlations. Thicker lines denote stronger connections. Of the 66 possible connections, 54 (81.82%) were nonzero, and the network density was 0.82, indicating that the variables were generally closely interconnected within this sample. Among these, 41 were positive correlations and 13 were negative correlations. From the perspective of community structure, the nodes are primarily clustered into 3 distinct communities based on theoretical constructs, including emotional intelligence (EI1-EI4), AI self-efficacy (AILS1-AILS4), and AI competence (AS1-AS4). Connections within each community are relatively dense, while cross-community connections are relatively sparse, which is largely consistent with the dimensional divisions of the NURSES framework. Stronger connections primarily occurred within dimensions rather than between them. The 3 strongest intradimensional connections were EI4 (emotional use)-EI3 (managing others’ emotions), AS3 (assessment)-AS1 (awareness), and AILS2 (anthropomorphic interaction)-AILS1 (assistance). The strongest interdimensional connection was between EI1 (emotional perception) and AILS1 (assistance). All edge weights (association strengths) are listed in Table 4.

**Table 4.** Marginal weights of emotional intelligence (EI), artificial intelligence (AI) self-efficacy (AILS), and AI literacy (AS) among nursing undergraduates.

EI1^a^	EI2^b^	EI3^c^	EI4^d^	AILS1^e^	AILS2^f^	AILS3^g^	AILS4^h^	AS1^i^	AS2^j^	AS3^k^	AS4^l^
EI1	0.00	0.34	0.20	0.28	0.22	0.11	0.00	0.05	0.00	–0.00	0.00	0.05
EI2	0.34	0.00	0.18	0.31	0.05	0.02	–0.02	0.07	–0.01	0.00	–0.01	0.02
EI3	0.20	0.18	0.00	0.44	0.00	–0.03	0.14	–0.07	0.00	0.00	0.00	–0.04
EI4	0.28	0.31	0.44	0.00	–0.12	–0.02	0.15	0.18	–0.01	0.00	0.00	–0.04
AILS1	0.22	0.05	0.00	–0.12	0.00	0.34	0.15	0.18	–0.01	0.00	–0.01	0.00
AILS2	0.11	0.02	–0.03	–0.02	0.34	0.00	0.33	0.22	0.00	0.00	–0.01	0.01
AILS3	0.00	–0.02	0.14	0.15	0.15	0.33	0.00	0.17	0.00	0.00	0.00	–0.03
AILS4	0.05	0.07	–0.07	0.18	0.18	0.22	0.17	0.00	0.03	–0.03	0.02	0.00
AS1	0.00	–0.01	0.00	–0.01	–0.01	0.00	0.00	0.03	0.00	0.00	0.66	0.15
AS2	–0.00	0.00	0.00	0.00	0.00	0.00	0.00	–0.03	0.00	0.00	0.46	–0.35
AS3	0.00	–0.01	0.00	0.00	–0.01	–0.01	0.00	0.02	0.66	0.46	0.00	–0.15
AS4	0.05	0.02	–0.04	–0.04	0.00	0.01	–0.03	0.00	0.15	–0.35	–0.15	0.00

a EI1: emotional perception.

b EI2: self-emotion management.

c EI3: managing others’ emotions.

d EI4: emotional application.

e AISL1: assistance.

f AISL2: anthropomorphic interaction.

g AISL3: comfort with artificial intelligence.

h AISL4: technical skills.

i AS1: awareness.

j AS2: usage.

k AS3: evaluation.

l AS4: ethics.

### Node Centrality Measures

**Figure 2.**
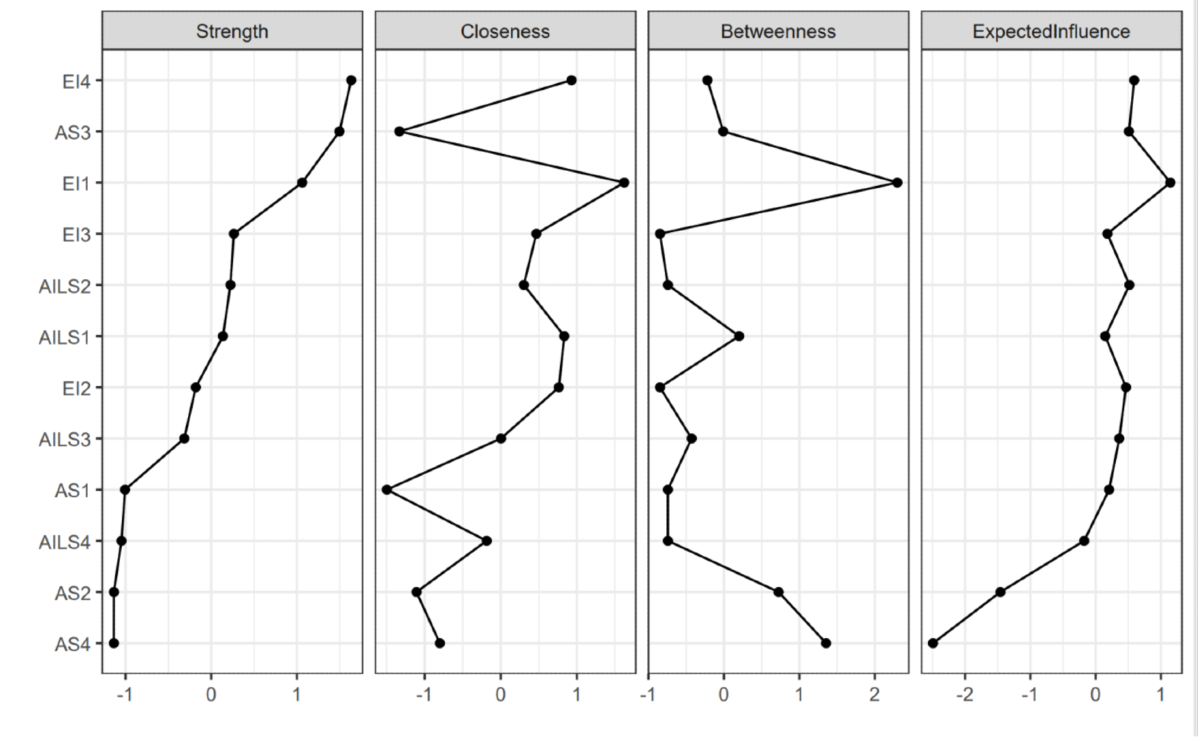
Standardized estimates of centrality measures for emotional intelligence, artificial intelligence (AI) self-efficacy, and AI literacy among nursing undergraduates. AILS1: assistance; AILS2: anthropomorphic interaction; AILS3: comfort with AI; AILS4: technical skills; AS1: awareness; AS2: usage; AS3: evaluation; AS4: ethics; EI1: emotional perception; EI2: self-emotion management; EI3: managing others’ emotions; EI4: emotional application.

**Figure 3.**
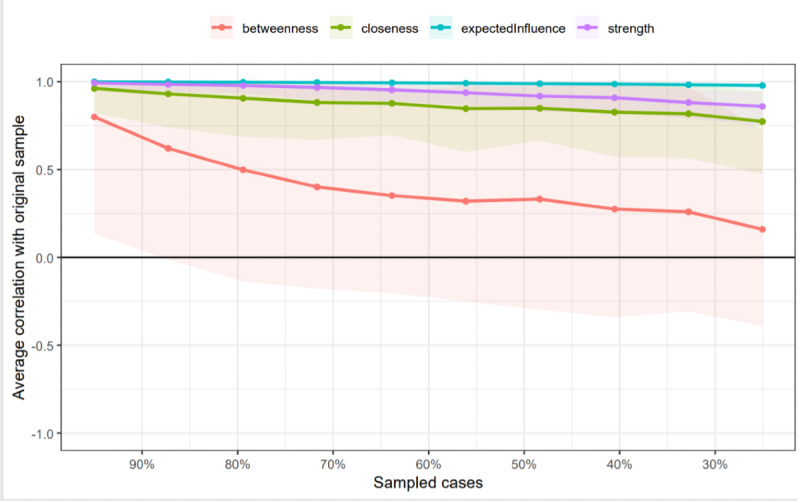
Correlation stability coefficients for node strength, betweenness, and closeness.

The standardized estimates of centrality indicators for factors influencing EI, AILS, and AS among nursing undergraduates are shown in Figure 2. Emotional use (EI4) and assessment (AS3) exhibit the highest strength centrality, indicating that these 2 variables showed the highest strength centrality, indicating they are highly connected within the network. Emotional perception (EI1) and ethics (AS4) exhibited the strongest mediating centrality, signifying their pivotal bridging role in network information transmission with potent moderating capacity; emotional perception (EI1) and awareness (AS1) also demonstrated the most prominent proximity centrality, reflecting their highest information transmission efficiency and accessibility within the network. Stability tests for network centrality metrics revealed correlation stability coefficients of 0.749, 0.749, and 0.361 for node strength, intermediary centrality, and closeness centrality, respectively (see Figure 3). This indicates robust stability for these centrality measures, with node strength demonstrating the highest stability.

### Edge Accuracy

**Figure 4.**
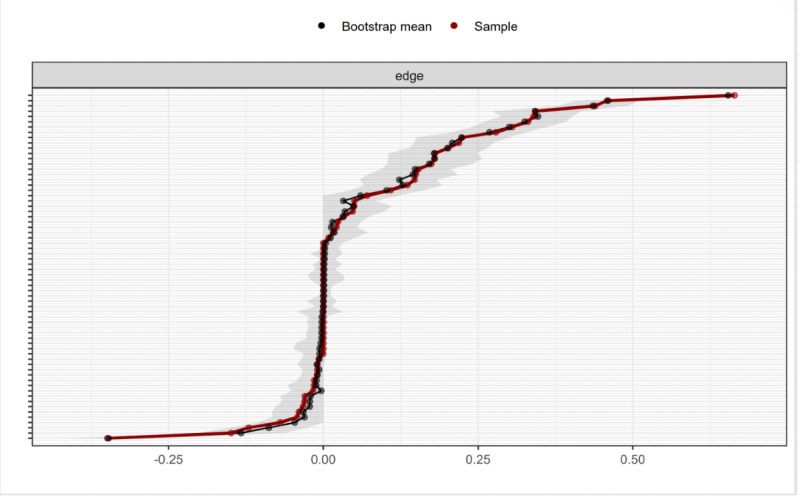
Guiding edge weights for emotional intelligence, artificial intelligence (AI) self-efficacy, and AI literacy among nursing undergraduates with 95% CIs.

The accuracy of edge weights was estimated using nonparametric bootstrapping. Results indicate that the 95% CIs for edge weights in the network are narrow, suggesting that the estimated edge weights in this study are highly precise (Figure 4).

### Node Bridge Strength

**Figure 5.**
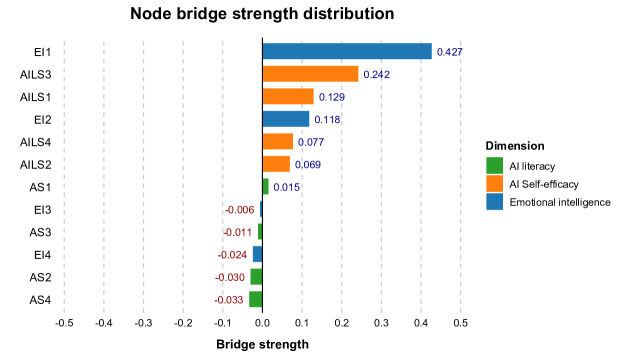
Depicts the bridge strength (z-score) of each node in the network. AILS1: assistance; AILS2: anthropomorphic interaction; AILS3: comfort with AI; AILS4: technical skills; AS1: awareness; AS2: usage; AS3: evaluation; AS4: ethics; EI1: emotional perception; EI2: self-emotion management; EI3: managing others’ emotions; EI4: emotional application.

Figure 5 displays the bridge strength for each node. EI1 “Emotion Awareness” (bridge strength=0.427) exhibits the highest bridge strength, followed by ALS3 “Comfort with AI” (bridge strength=0.242), ALS1 “Assistance” (bridge strength=0.129), and EI2 “Self-Emotion Management” (bridge strength=0.118), suggesting they may serve as bridging nodes in this network.

## Discussion

### Principal Findings

This study is the first to use network analysis methods within the NUR.S.E.S. framework to examine the associations among EI, AILS, and AS among undergraduate nursing students. The network analysis identified that emotion use (EI4) and assessment ability (AS3) showed the highest strength centrality, indicating their prominent positions within the network. Emotion perception (EI1) had the highest bridge strength, suggesting it may serve as a connecting node between the EI domain and the AILS and AS domains. These findings provide insights into the psychological mechanisms underlying the development of AS among nursing students, offer a concrete explanation of how the 6 dimensions of the NURSES framework interact at the psychological level, and provide a critical foundation for the future development of targeted, comprehensive interventions that address the full spectrum from emotional foundations to technical confidence.

### Overall Characteristics of EI, AILS, and AS

This study conducted a descriptive analysis of nursing students’ EI, AILS, and AS. Results indicate that undergraduate nursing students’ AILS (mean 4.68, SD 0.78) and AS (mean 4.60, SD 0.59) were generally at a moderately high level, while EI (mean 3.57, SD 0.55) scored relatively low. This situation highlights a key phenomenon: nursing students possess strong confidence in their ability to learn and master AI technologies and have established a foundational level of AS. However, the development of their EI has not kept pace, consistent with the findings of Bewersdorff et al [38]. From a specific-dimension perspective, within AILS, the “Comfort Level with Artificial Intelligence (AILS3)” dimension scored the highest (mean 4.96, SD 0.98), indicating that nursing students experience relatively positive feelings when interacting with AI. This finding aligns with the research results of Volpato et al [48]. In terms of EI, the “Emotional perception (EI1)” dimension had the lowest mean score 3.41 (SD 0.54), suggesting that nursing students have significant room for improvement in their ability to accurately identify and understand their own and others’ emotions. This weakness may directly impact nursing students’ empathetic communication and emotional regulation in high-pressure clinical settings, posing a potential risk to patient safety; failure to promptly identify patients’ anxiety or distress may delay nursing decisions.

### Emotion Perception: the Psychological Bridge Connecting the Emotional World and the AI World

The network structure constructed in this study showed that emotional perception (EI1), defined as the ability to recognize one’s own and others’ emotional states, was strongly connected to AILS and AS nodes. In this sample, emotional perception had the highest bridge strength, indicating that it was relatively well-connected to nodes in other communities. This pattern is consistent with Fu’s [49] finding that emotional competence is associated with mental health and with deeper engagement with AI in educational contexts. The high bridge strength suggests that the ability to recognize emotions may be associated with trust in AI technology, which aligns with previous research showing that individuals with higher EI tend to monitor emotional cues and manage stress effectively [50]. Specifically, the positive connection between emotional perception and the assistance dimension (AILS1) indicated that students who reported greater awareness of their own confusion, anxiety, or sense of accomplishment during technology learning were also more likely to view AI as a helpful tool [51]. This pattern is consistent with the observation by Mosleh et al [35]. that AI integration in teaching creates opportunities for emotional recognition and regulation, which may be easier for students with stronger emotional perception.

### Emotional Application and Evaluation: the Engine Driving the Development of AS

The centrality measures showed that emotional use (EI4) and assessment (AS3) had the highest strength centrality, indicating that they were highly connected nodes within the network [52]. This pattern suggests that AS is associated not only with cognitive factors but also with emotional factors. Emotional use, defined as the ability to use emotional information for planning, creativity, and problem-solving, was positively connected with critical evaluation of AI outputs [13]. This finding is consistent with the observation that some individuals turn to GenAI for emotional guidance [50] and that emotional use may help nursing students engage in critical thinking when faced with complex AI outputs [53]. Evaluation ability, which concerns the judgment of AI-generated content [33], was closely connected with emotional use in the network. This pattern aligns with the finding by Deng and Chen [19] that AI-driven environments can support cognitive flexibility and independent judgment, possibly through emotional resilience. These exploratory observations invite further investigation using longitudinal or experimental designs to examine whether and how emotional use and evaluation skills might be related to AS [54].

### AILS: a Potentially Important Bridging Node Between Emotion and AS

Network analysis results showed that EI, represented by emotional perception (EI1), had a positive connection with AILS, represented by assistance (AILS1) and comfort with AI (AILS3). For example, the edge weight between EI1 and AILS1 was 0.22. AILS also showed positive connections with AS, represented by usage (AS2) and evaluation (AS3). This pathway pattern, in which EI connected to AILS and AILS in turn connected to AS, is consistent with a mediating structure [55]. However, due to the cross-sectional design, this pattern should be interpreted as exploratory. It suggests that AILS may occupy a bridging position, but formal mediation analysis using longitudinal data would be required to test this hypothesis [49]. Prior research has shown that EI is associated with self-efficacy and with cognitive engagement with AI [49]. The connection pattern observed here is compatible with the idea that confidence in technical skills (AILS4) is associated with more frequent use of AI tools (AS2) [56]; that comfort with AI (AILS3) and anthropomorphic interaction (AILS2) are associated with lower anxiety and more exploration [25]; and that the belief that AI serves as an effective assistant (AILS1) is associated with reduced operational anxiety [53]. These observations are hypothesis-generating and require replication in independent samples with stronger designs. The significance of this approach for clinical practice lies in the fact that only by simultaneously strengthening both the emotional foundation and technical confidence can knowledge be translated into safe and effective clinical practice.

### The Deep Mechanisms of AS Revealed by Negative Correlations

The most significant negative correlation was found between “Ethics (AS4)” and “Usage (AS2)” in the network. This indicates that at the current stage, nursing students who are more concerned about AI ethical issues (such as data privacy) may be more cautious in their use of AI tools, even exhibiting lower usage frequency [57]. Although this contradicts findings showing positive correlations between EI and reasons for adopting AI services or adoption/usage intentions [58], it reflects the development of critical thinking. When nursing students recognize the potential risks of technology, they may transition from blind tool users to critical thinkers, adopting more rational, selective use behaviors [59]. This finding resonates with the perspective of Ricon [60] that premature or excessive enthusiasm in medical AI may obscure ethical concerns, while measured caution is a prerequisite for responsible innovation. Participants in one study expressed concerns about potential privacy breaches and the risk of personal data theft [61]. Therefore, educators should not pursue unrestricted usage frequency but instead focus on cultivating students’ high-level application skills grounded in strong ethical vigilance.

Second is the negative correlation between “Emotional Intelligence in Others (EI3)” and “Anthropomorphic Interaction (AILS2).” This negative correlation may reflect multiple mechanisms. First, individuals with high EI are able to clearly distinguish the fundamental differences between human emotional connections and AI-simulated interactions. The ability to manage others’ emotions manifests as the capacity to perceive, influence, and regulate others’ emotions in interpersonal interactions, whereas the tendency toward anthropomorphic interaction is a psychological inclination to ascribe human characteristics to AI and interact with it in a social manner. Nursing students with high EI have a deeper understanding that, although AI can simulate empathetic responses, it lacks genuine emotional intent and reciprocity. Consequently, they consciously avoid viewing AI as an emotional substitute to prevent the development of inappropriate emotional dependence, which is consistent with Dakakni and Safa [62]. Second, cultural factors may play a role. In the context of Eastern culture, which emphasizes interpersonal harmony and emotional connection, nursing education places a high value on “authentic interpersonal interaction.” Nursing students with high EI may have internalized these professional values, leading them to adopt a more cautious or even resistant attitude toward anthropomorphizing AI. Third, from the perspective of clinical practice, this negative correlation carries positive ethical implications. The core of nursing practice lies in establishing trust within therapeutic interpersonal relationships. If nursing students are overly inclined to anthropomorphize AI and blur the boundaries between humans and machines, it may diminish their sensitivity to the emotional needs of real patients. Therefore, the lower tendency toward anthropomorphic interaction exhibited by nursing students with high EI can be viewed as an adaptive strategy for upholding the core values of the nursing profession [63].

### System Integration Under the NUR.S.E.S. Framework: Synergistic Development of EI, AILS, and AS

The findings of this study provide empirical support for the NUR.S.E.S. framework, revealing the underlying psychological mechanisms behind its 6 dimensions. Specifically, emotional perception (EI1), as the strongest bridge node, empirically demonstrates the central role of E (EI) and S (social-emotional comfort) as the emotional cornerstones within the framework [64]; emotional application (EI4) and assessment (AS3), as core driving nodes, correspond to E and R (defect identification), respectively, indicating that emotional competence and technical critical thinking share an intrinsic synergistic mechanism; the various dimensions of AILS occupy key positions in the network that connect emotion and cognition, validating the theoretical premise within the framework that S (AILS) serves as the core driving force [65]. Ultimately, the synergy of all these elements achieves the framework’s ultimate goal—S (shape the future)—that is, shaping the future [27].

In addition, this study provides an initial exploratory mapping of the interrelated factors associated with AS among nursing undergraduates, and these findings may offer insights for future research. From a practical perspective, nodes with high centrality, such as emotional use and evaluation, could exert influence on the overall network configuration through their connections with other nodes. Bridge nodes, such as emotional perception and comfort with AI, may transmit influences across different domains, making them potential targets for hypothesis-driven interventions. Accordingly, nursing educators might consider several tentative strategies, fostering students' emotional perception skills, which appear to link EI with AILS; creating low-anxiety AI learning environments to enhance comfort with AI; helping students set personalized learning goals and reflect on their emotional responses to strengthen emotional use and critical evaluation; and introducing case-based learning that combines clinical scenarios with AI outputs to ground technical critique and ethical decision-making in empathy for patients’ emotional needs. Such a multidimensional approach, addressing emotional, cognitive, and skill components simultaneously, may support the development of nursing students as future professionals who can shape AI integration in nursing practice. However, all suggestions require empirical validation through longitudinal or experimental designs.

### Research Significance, Limitations, and Future Directions

This study is the first to visualize, through network analysis, the patterns of association among EI, AILS, and AS in nursing students. It identified nodes that showed high centrality and bridging strength (emotional perception, emotional application, evaluation, comfort with AI). The observed association pattern suggests that emotional competence may be closely linked with technological confidence, which in turn may be linked with AS. These exploratory findings offer preliminary clues for future hypothesis-driven research. However, they do not establish causality, nor do they directly identify effective intervention targets. Any educational implications drawn from these results are speculative and require empirical validation [56].

This study has several limitations. First, the cross-sectional design cannot establish causal relationships between variables. Although the network pattern is consistent with some causal pathways, a reverse pathway (eg, higher AS leading to greater AILS) is equally plausible. Future research should use longitudinal or intervention designs to test dynamic relationships. Second, the sample originated from a single university, limiting generalizability. Cross-regional and cross-cultural comparisons are needed. Third, self-report scales may introduce bias; future studies could incorporate behavioral data and qualitative interviews.

Despite these limitations, this study offers profound theoretical insights and precise practical guidance for cultivating a new generation of nursing professionals who are “technically proficient, confident, and emotionally intelligent” in the era of AI. Future education and research should focus on developing integrated intervention programs that combine emotional education with technical training, empowering nursing students to embrace the challenges of the intelligent health care era fully.

## Abbreviations

AI: artificial intelligence
AILS: artificial intelligence self-efficacy
ANA: American Nurses Association
AS: AI literacy
CFI: comparative fit index
CS: correlation stability
EI: emotional perception
GenAI: generative artificial intelligence
RMSEA: root-mean-square error of approximation
SRMR: standardized root-mean-square residual
TLI: Tucker-Lewis index
